# Application of metagenomic next-generation sequencing as an adjunct to conventional microbiological testing for the diagnosis of infection in kidney transplant recipients

**DOI:** 10.3389/fcimb.2026.1713707

**Published:** 2026-07-17

**Authors:** Hanilen Vuth, Weili Wang, Wenhan Qin, Meng Yang, Youzan Li, Qiang Zhou, Xiaosong Xu, Juan Zhang, Hongwen Zhao

**Affiliations:** 1 Department of Nephrology, the First Affiliated Hospital of Army Medical University, Chongqing, China; 2 Department of Nephrology, Preah Ket Mealea Hospital., Phnom Penh, Cambodia; 3 Infectious Diseases Department, the First Affiliated Hospital of Army Medical University, Chongqing, China

**Keywords:** drug resistance genes, kidney transplantation, metagenomic next generation sequencing(mNGS), pulmonary infection, urinary tract infections

## Abstract

**Background:**

Kidney transplant recipients are highly susceptible to opportunistic and nosocomial infections that demand rapid and accurate diagnosis due to the broad and complex spectrum of pathogens. Conventional microbiological testing (CMT) is often limited, particularly when patients are already receiving antimicrobial therapy at the time of sampling. This study aimed to evaluate the clinical value of metagenomic next-generation sequencing (mNGS) as a complementary diagnostic approach to CMT, with a focus on concordance and discrepancies between the two methods across peripheral blood, sputum, bronchoalveolar lavage fluid (BALF), and urine samples.

**Methods:**

We conducted a retrospective study of kidney transplant recipients with suspected infections who underwent simultaneous mNGS and CMT testing between March 2022 and May 2024. The impact of prior antibiotic exposure on diagnostic yield was assessed. Detection of antimicrobial resistance (AMR) genes by mNGS and subsequent modifications in anti-infective management were also analyzed.

**Results:**

A total of 243 samples (57 blood, 96 sputum, 71 BALF, 19 urine) were included. Across all sample types, mNGS demonstrated significantly higher positive rates than CMT (blood: 78.95% vs 21.05%; BALF: 90.14% vs 19.72%; sputum: 92.71% vs 20.83%; urine: 89.47% vs 36.84%; all P<0.001). Prior antibiotic exposure markedly reduced CMT positivity but had minimal impact on mNGS detection. Concordance analysis showed 40.35% of samples were positive by both methods, while 60.1% were negative by CMT but positive by mNGS. In addition to pathogens identified by CMT, mNGS detected a broader range of microorganisms, including viruses (e.g., cytomegalovirus, Epstein-Barr virus, SARS-CoV-2), fungi (Pneumocystis jirovecii), and parasites (Strongyloides stercoralis, Toxoplasma gondii). Overall, mNGS-guided results refined antibiotic treatment strategies in 110 cases (60.11%).

**Conclusion:**

mNGS serves as a valuable adjunct to CMT in kidney transplant recipients, providing rapid and comprehensive pathogen identification. However, from a health economics perspective, mNGS should be applied selectively according to clinical needs, rather than as a universal first-line diagnostic method.

## 1 Introduction

Kidney transplant is still the optimal treatment that offers a survival benefit and quality of life for patients with end stage kidney disease compared to dialysis (Ding et al., 2024). Despite its benefits, the long term use of immunosuppressants significantly increases the risk of infection in kidney transplant recipients, leading to an increase in the incidence of kidney rejection and graft loss, which affects survival rate of recipients (Yeşiler et al., 2022).

In current clinical practice, microbiological testing remains the primary method for providing clinicians with a definitive basis for the diagnosis and guiding treatment of infections. Therefore, early and accurate identification of causative pathogens is essential for effective clinical management, particularly in immunocompromised populations such as kidney transplant recipients (Gadsby and Musher, 2022; Oliveira et al., 2023).

Conventional microbiological testing (CMT), including microbial culture, smear microscopy, staining, and polymerase chain reaction (PCR), remains widely used in clinical practice (Laga, 2020; Miller et al., 2024). These methods primarily rely on microscopic visualization or *in vitro* cultivation of pathogens from clinical sample. However, early pathogen detection remains challenging due to the limited sensitivity of these techniques, the requirement for specific primers, prolonged turnaround times, and their inability to simultaneously detect multiple pathogens. Chronic immunosuppression in kidney transplant recipients predisposes them not only to bacterial infections but also to a spectrum of opportunistic fungi, viruses, and even parasites. These constraints delay accurate pathogen identification and treatment, highlighting an urgent need for rapid, broad range molecular or metagenomic approaches that do not rely on predefined targets and can improve early detection and clinical outcomes (Gu et al., 2019; Hao et al., 2023).

Metagenomic next generation sequencing (mNGS) is an unbiased, untargeted diagnostic approach that enables direct detection of pathogens from a variety of clinical samples, including bronchoalveolar lavage fluid (BALF), peripheral blood, sputum, urine, and cerebrospinal fluid (Wu et al., 2024). Compared to conventional methods, mNGS has emerged as a promising tool due to its rapid turnaround time and broad detection spectrum, allowing simultaneous identification of bacteria, fungi, viruses, parasites, atypical organisms, and even novel pathogens through DNA or RNA analysis. Its high diagnostic yield, reduced susceptibility to prior antibiotic exposure, and ability to lower rates of delayed or missed diagnoses contribute to more timely and targeted treatment. This may also reduce inappropriate antibiotic use and help combat antimicrobial resistance (Ye et al., 2024). BALF is widely considered the optimal sample type for diagnosing pulmonary infections due to its high sensitivity (Davidson et al., 2020; Yang et al., 2022). Currently, the diagnostic utility of mNGS has been increasingly evaluated across diverse clinical contexts, including lung transplant recipients, individuals with HIV infection, and patients with central nervous system (CNS) infections (Ju et al., 2022; Benoit et al., 2024; Sun et al., 2024).

However, previous studies focusing specifically on its performance in kidney transplant recipients with pneumonia and urinary tract infection remain limited, with most evidence derived from case reports or small-scale studies. Therefore, this study aimed to assess the diagnostic performance of mNGS applied to BALF, blood, sputum, and urine samples in kidney transplant recipients presenting with sepsis, pulmonary, or urinary tract infections, compared with conventional microbiological methods. Furthermore, the findings of this study provide new insights into the management of infections in kidney transplant recipients during hospitalization.

## 2 Methods and materials

### 2.1 Study design and samples

This was a single center retrospective study from the first affiliated hospital of army medical university, Chongqing, China. The study adhered to the ethical principles outlined in the Declaration of Helsinki and received approval from the Ethics Committee of the First Affiliated Hospital of army medical university (Approval No. (B)KY2025115).

**Figure 1:**
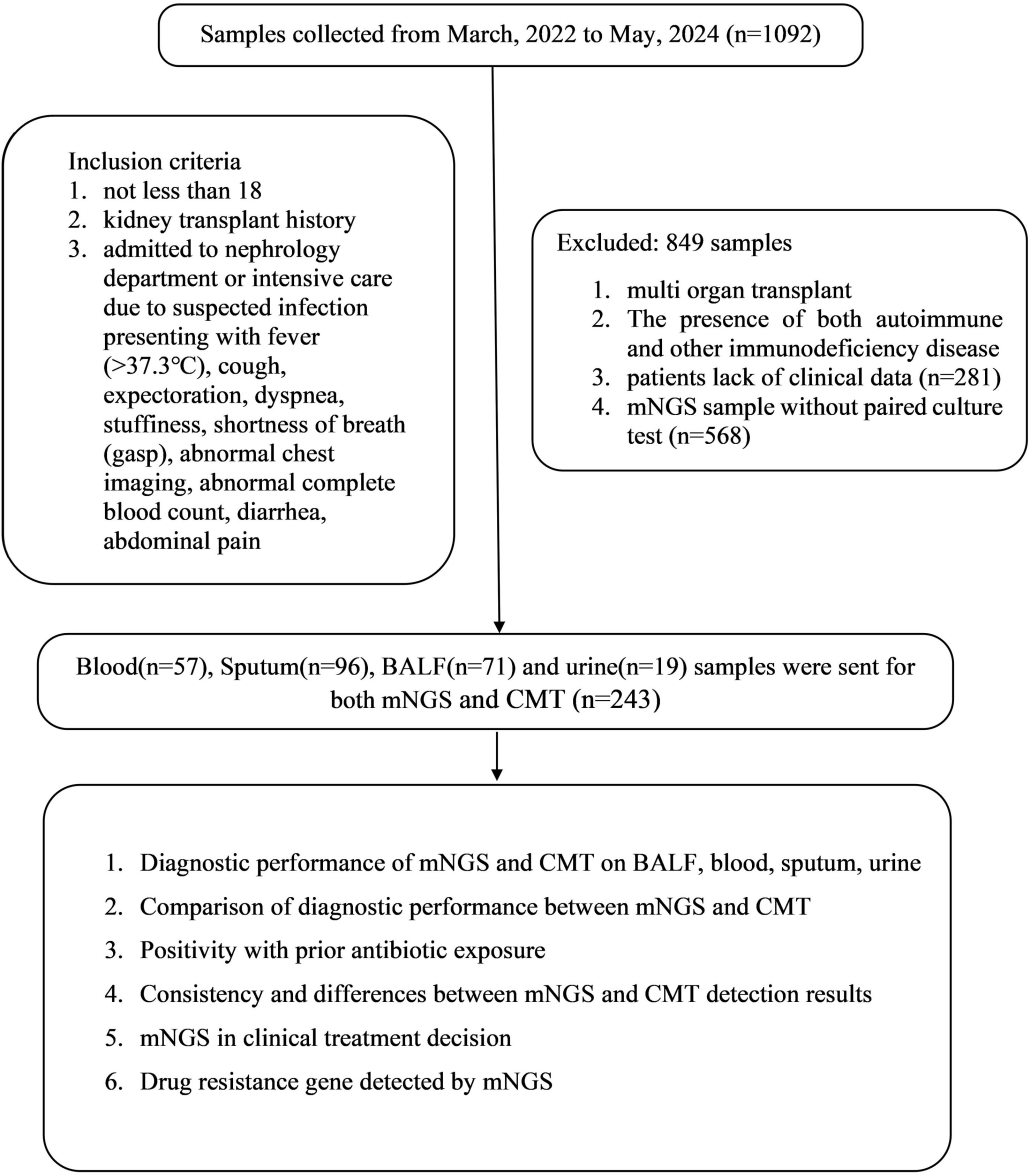
Flowchart of sample selection.

The data of recipients who hospitalized from March 2022 to May 2024 were enrolled. Both mNGS and CMT were concurrently applied to BALF, peripheral blood, and sputum samples to facilitate comparative pathogen detection. The inclusion criteria were as follows: 1. kidney transplantation recipients; 2. recipients were diagnosed or suspected infection, including exhibiting fever (>37.2 °C), cough, expectoration, dyspnea, stuffiness, shortness of breath, abnormal chest imaging, abnormal complete blood count, discomfort during urination, etc. The exclusion criteria were as follows:1. the clinical data of recipients was absent; 2. multi organ transplant; 3. mNGS sample without paired conventional microbiological testing; 4. mNGS sample failed to pass quality control. The flowchart of sample selection was display in **Figure 1**.

### 2.2 mNGS analysis

For blood samples, at least 4 mL of whole blood was centrifuged at 1600 g for 10 min, followed by a second centrifugation of the supernatant at 16,000 g for 10 min to obtain plasma. For BALF, sputum and urine samples were collected under aseptic conditions. A 0.6 mL aliquot of each sample was placed in a 1.5 mL microcentrifuge tube with 1.0 g of 0.5-mm glass beads and agitated at 3200 rpm for 30 min using a horizontal vortex mixer. From this, 0.2 mL was used for DNA extraction with the TIANamp Micro DNA Kit (Tiangen Biotech), according to the manufacturer’s instructions. DNA libraries were prepared via fragmentation, end repair, adapter ligation, and PCR amplification, and sequenced on the Illumina NextSeq 550 platform using 75 bp single-end reads.

### 2.3 Statistical analysis

All statistical analyses were performed using the statistical package SPSS for windows version 22.0 (IBM Corporation Armonk, NY, USA). Results were listed as mean ± standard deviation (SD) for continues variables. Continues variables were compared using student’s unpaired T-test. Counting data were expressed as the number of cases with percentage (%). The chi squared test was conducted to evaluate the detection rate between mNGS and CMT. Categorical variables were compared with Pearson’s x^2^ test or fisher’s exact test. Statistical tests were two tailed and statistical significance was defined as *P* < 0.05.

## 3 Results

### 3.1 Recipients characteristic

Details of the clinical characteristics of the enrolled kidney transplant recipients were analyzed. A total of 243 samples were collected during different phases of infection, including 71 BALF samples, 96 sputum samples, 57 peripheral blood samples, and 19 urine samples. The clinical features including demographic characteristics, laboratory findings, comorbidities, signs of infection, prior antibiotic exposure, transplantation details, dialysis history, and immunosuppressant use are summarized in **Table 1**.

**Table 1:** Characteristics of included recipients.

Demographics	Total (n = 243)
Age, years (mean ± SD)	40.79 ± 11.80
Sex (Male/Female, %)	192/51 (79.01%)
BMI	
<18.5kg/m^2^	32 (13.17%)
18.5kg/m^2^≤BMI<24.0kg/m^2^	144 (59.26%)
24.0kg/m^2^≤BMI<28.0kg/m^2^	54 (22.22%)
≥28.0kg/m^2^	13 (5.35%)
Post-transplantation time, years (mean ± SD)	3.83 ± 2.39
Less than 1 year	0
1–3 years	133 (54.73%)
More than 3 years	110 (45.27%)
Type of transplant	
Deceased donor	218 (89.71%)
Living donor	25 (10.29%)
Pre transplantation Dialysis form	
Hemodialysis	98 (40.33%)
Peritoneal dialysis	48 (19.75%)
Hemodialysis+ Peritoneal dialysis	3 (1.23%)
Unknown	94 (38.68%)
Pre transplantation Dialysis duration (month)	
Less than 1 year	29 (11.93%)
1–5 years	126 (51.85%)
More than 5 years	4 (1.65%)
Unknown	84 (34.57%)
Immunosuppressant	
Tacrolimus + MMF+ Pred	113(46.5%)
Tacrolimus + MPS + Pred	40(16.46%)
Tacrolimus + MZR + Pred	9(3.7%)
Tacrolimus + mTORi + MMF+ Pred	6(2.47%)
Tacrolimus + mTORi + MPS + Pred	15(6.17%)
CsA + MMF+ Pred	18(7.41%)
CsA + MPS + Pred	8(3.29%)
CsA + MZR + Pred	2(0.82%)
CsA + mTORi + MMF+ Pred	6(2.47%)
CsA + mTORi + MPS + Pred	3(1.23%)
CsA + mTORi + MZR + Pred	4(1.65%)
Unknown	19(7.82%)
Comorbidities	
Hypertension	177 (72.84%)
Diabetes	19 (7.82%)
Hyperuricemia	6 (2.47%)
Heart disease	4 (1.65%)
COPD	0
More than one comorbidity	76 (31.27%)
Symptoms	
Fever	150 (67.73%)
Cough	169 (69.55%)
Dyspnea	17 (6.99%)
Fatigue	46 (18.93%)
Diarrhea	26 (10.7%)
Abdominal pain	10 (4.12%)
Dysuria	11(4.53%)
Outcome	
Days from onset to admission (mean ± SD)	9.65 ± 23.33
Respiratory failure	40 (16.46%)
Days of admission (mean ± SD)	26.48 ± 22.71
Require ICU admission	40 (16.46%)
Days of ICU stay (mean ± SD)	37.3 ± 32.42
All-cause death	1 (0.41%)
Perform mNGS of recipients who without significant improvement after undergoing CMT	179 (73.6%)
Perform mNGS of recipients whose condition worsened after undergoing CMT	64 (26.74%)
The days of performing mNGS after CMT (mean ± SD)	3.22 ± 2.81
Prior antibiotic exposure	183 (75.81%)
Treatments	
Antibacterial drugs	39 (16.05%)
Antiviral drugs	11 (4.53%)
Antifungal drugs	7 (2.88%)
Antibacterial + Antifungal drugs	103 (42.39%)
Antibacterial + Antiviral drugs	12 (4.94%)
Antibacterial + Antifungal + Antiviral drugs	71 (29.22%)

Data were provided as n/percentage (%) or mean ± standard deviation. COPD, chronic obstructive pulmonary disease; Tac, Tacrolimus; MMF, Mycophenolate Mofetil; MPS, Mycophenolate Sodium; MZR, Mizoribine; mTORi, Mammalian Target of Rapamycin Inhibitors; CsA, Cyclosporin A; Pred, Prednisone.

Among the enrolled recipients, there were 192 males and 51 females, with a mean age of 40.8 ± 11.8 years. Most recipients received grafts from deceased donors (89.71%). Fever was documented in 150 (67.73%), while cough was the most frequent symptom, reported in 169 (69.55%). Hypertension (177, 72.84%) and diabetes (19, 7.82%) were the most common comorbidities, and 76 (31.27%) had more than one comorbidity. Hemodialysis was the predominant pre-transplant modality (98, 40.33%), and 126 (51.85%) had undergone dialysis within the preceding five years. At the time of admission, 133 (54.73%) were within three years post-transplant, while 110 (45.27%) were more than three years post-transplant. 40 (16.46%) developed acute respiratory distress syndrome (ARDS) requiring intensive care unit (ICU) admission, with one mortality (0.41%). The mean length of hospital stay for all recipients was 26.48 ± 22.71 days. In contrast, recipients who required ICU admission had an ICU stay of 37.3 ± 32.42 days. In most recipients (179, 73.6%), the mNGS was performed in recipient who did not show significant improvement after undergoing CMT. In 64 recipients (26.74%), mNGS was performed due to clinical deterioration following CMT guided management. The interval between CMT and mNGS testing was 3.22 ± 2.81days.

All recipients received standard immunosuppressive therapy based on clinical conditions within the therapeutic range to ensure efficacy and safety including prednisone, calcineurin inhibitors (tacrolimus or cyclosporine) and mycophenolate mofetil (MMF) or mycophenolate sodium (MPS), with or without mammalian target of the rapamycin inhibitors (mTORi).

### 3.2 Diagnostic performance of mNGS on BALF, sputum, blood, urine

Among the 243 enrolled samples, we identified a total of 457 pathogens by mNGS encompassing 50 distinct species including 20 types of bacterial, 9 types of fungi, 17 types of viruses, and 4 types of parasites.

In 57 peripheral blood samples, mNGS detected 3 bacterial, 3 fungal, 8 viral, and 2 parasitic species, with the top five were Torque Teno Virus (TTV, 16, 28.1%), human herpesvirus 5 (CMV, 10, 17.54%), herpes simplex virus 1 (HSV-1, 5, 8.77%), Enterococcus faecalis (5, 8.77%), and SARS-CoV-2 (4, 7.02%).

In 96 sputum samples, 10 bacterial, 6 fungal, 10 viral, and 3 parasitic species were identified, Which the top 5 were TTV (86, 89.58%), CMV (49, 51.04%), human herpesvirus 7 (HHV-7, 20, 20.83%), Epstein-Barr virus (EBV, 16, 16.67%), HSV-1 (12, 12.5%).

BALF samples yielded 18 types of pathogens, totaling 68 detections, with the top five being SARS-CoV-2 (10, 14.08%), Aspergillus fumigatus (9, 12.68%), Candida albicans (7, 9.86%), Klebsiella pneumoniae (7, 9.86%), and Enterococcus faecium (6, 8.45%).

In 19 urine samples, 6 bacterial, 2 fungal, and 2 viral species were identified, the top 5 pathogens were BK polyomavirus (BKV, 14, 73.68%), Escherichia coli (13, 68.42%), Enterococcus faecium (10, 52.63%), JC polyomavirus (JCV, 8, 42.11%), and Enterococcus faecalis (7, 36.84%).

**Figure 2:**
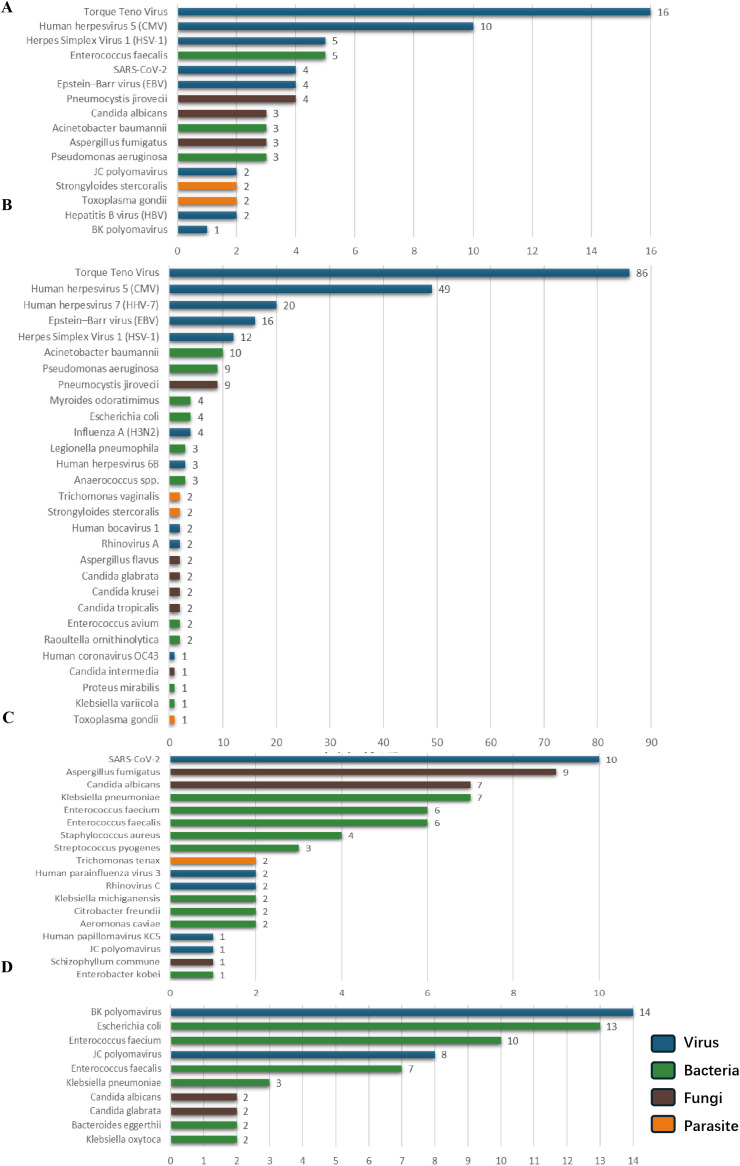
Pathogens detected by mNGS among different sample types. **(A)** blood; **(B)** Sputum; **(C)** BALF; **(D)** Urine.

Among the four sample types analyzed by mNGS, Sputum samples had the highest detection rate. Frequently identified pathogens across all sample types included TTV, CMV, JCV, BKV, Pneumocystis jirovecii, and Aspergillus fumigatus. These organisms are clinically significant in this population, being strongly associated with pulmonary infections, urinary tract infections, and immunosuppression related complications. JCV was detected in blood, BALF, and urine while TTV, CMV, EBV, and HSV-1 were found in both blood and sputum. Regarding Opportunistic fungi, including Aspergillus species was detected in blood, sputum and BALF whereas Pneumocystis jirovecii was detected in blood and sputum, highlighting their clinical relevance in immunocompromised hosts. Acinetobacter baumannii and Pseudomonas aeruginosa were found in blood and sputum while Klebsiella pneumoniae was identified in BALF and urine. Enterococcus species and Candida species were the only genera consistently detected in all four sample types. Importantly, all pathogens were also detected in blood, suggesting that blood may serve as a practical alternative diagnostic sample when respiratory or urine samples are not readily available (**Figure 2**).

### 3.3 Diagnostic performance of conventional microbiological testing on BALF, sputum, blood, urine

**Figure 3:**
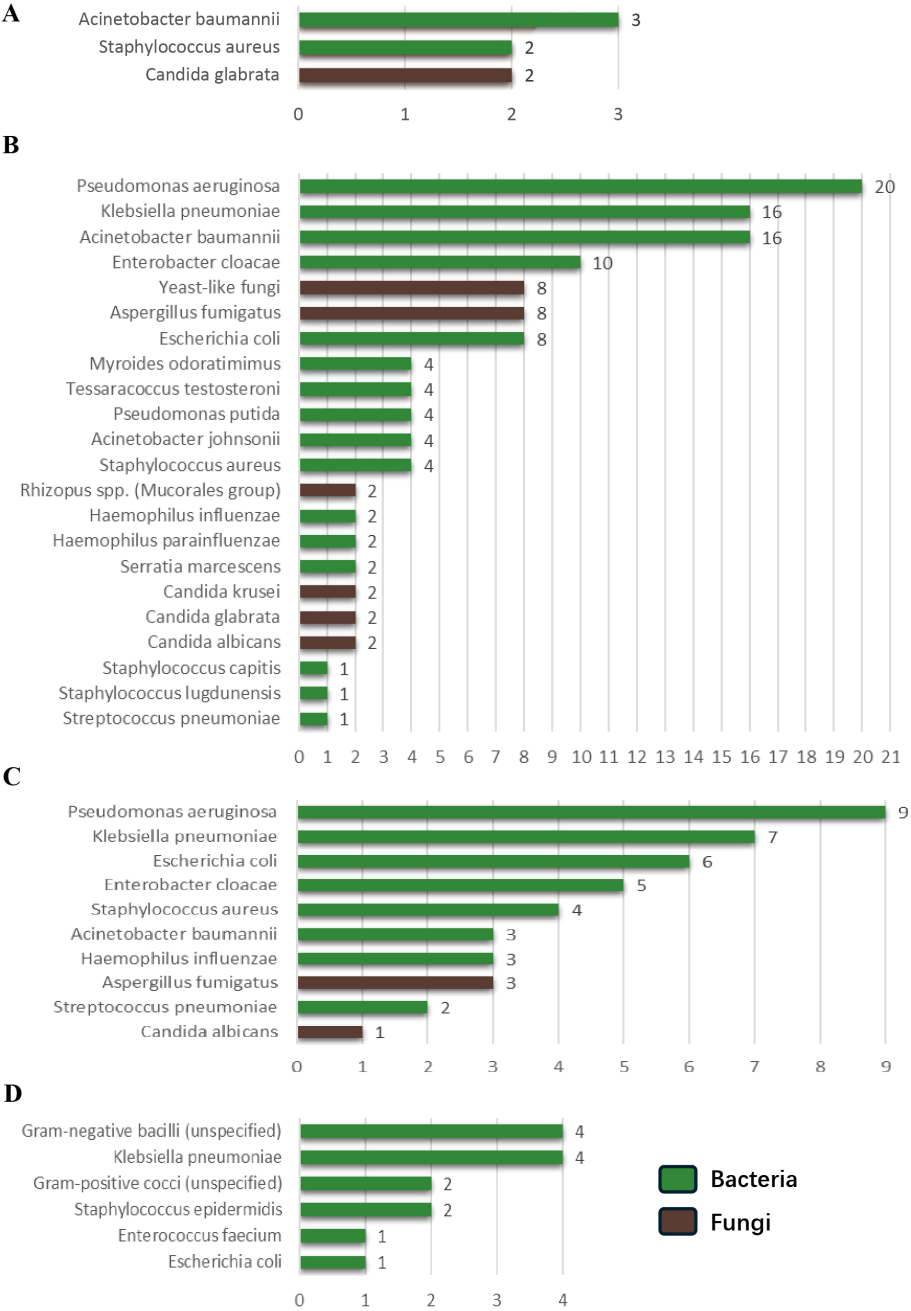
Pathogens detected by CMT among different sample types. **(A)** blood; **(B)** Sputum; **(C)** BALF; **(D)** Urine.

CMT across the four sample types identified a total of 187 pathogens, comprising 159 bacterial and 28 fungal isolates. The most frequently isolated bacterial species were Pseudomonas aeruginosa (n=29*)*, Klebsiella pneumoniae (n=27), Acinetobacter baumannii (n=22), and Escherichia coli (n=15). Fungal pathogens were detected predominantly in sputum (25%), followed by BALF (5.63%). The identified fungi included Aspergillus fumigatus, Candida albicans, Candida glabrata, Candida krusei, Rhizopus species and other yeast-like fungi. Notably, no viral or parasitic organisms were detected by CMT in any sample type, which indicates the deficiency of CMT compared with mNGS. The distribution by sample is presented in **Figure 3**.

### 3.4 Comparison of diagnostic performance between mNGS and CMT

Using a composite clinical diagnosis as the reference standard, all samples were analyzed by both of mNGS and CMT. Across all sample types, mNGS exhibited a significantly higher positivity rate compared to CMT with 78.95% vs 21.05%, (*P* < 0.001) in blood sample, 90.14% vs 19.72%, (*P* < 0.001) in BALF sample, 92.71% vs 20.83%, (*P* < 0.001) in Sputum sample, and 89.47% vs 36.84%, (*P* < 0.001) in Urine sample. Sputum samples yielded the highest mNGS positivity rate (92.71%), while urine had the highest positivity rate by CMT (36.84%).

**Figure 4:**
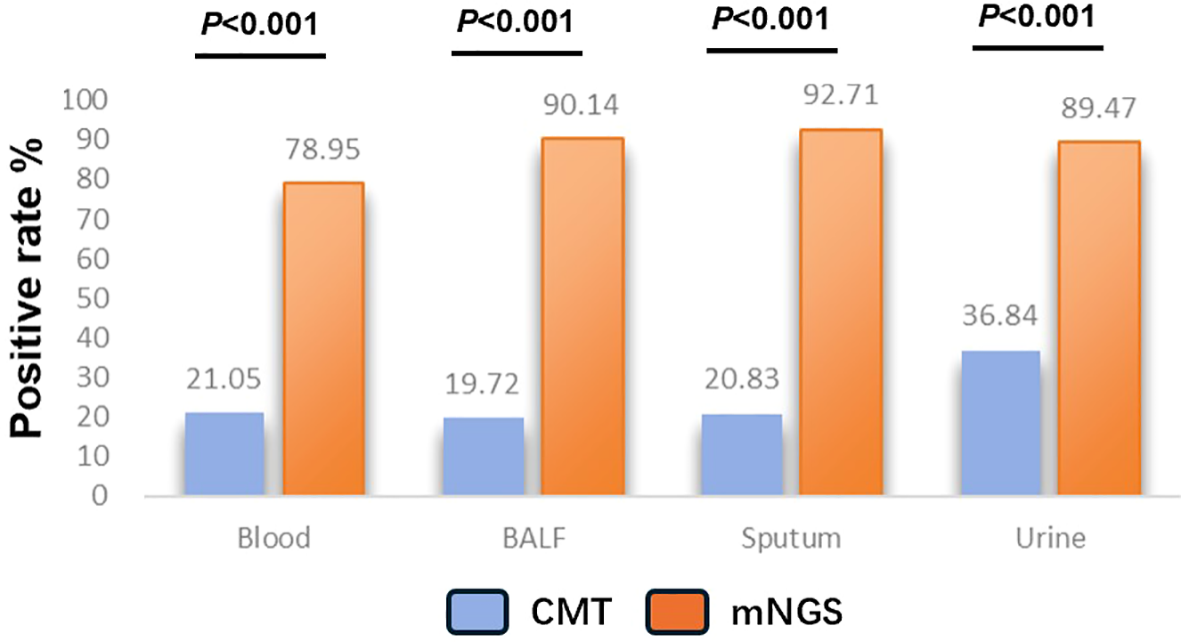
Comparison of positive rate between CMT and mNGS.

CMT achieved its highest detection rates in bacterial (65.43%) and fungal (11.52%) infections. In contrast, mNGS demonstrated bacterial and fungal detection rates of 49.38% and 20.16%, respectively, while also offering the added advantage of identifying viral and parasitic organisms. Notably, most pathogens detected by CMT were also captured by mNGS. Importantly, mNGS demonstrated superior performance in detecting multiple pathogens infections and rare pathogens that were undetectable by conventional methods, thereby offering enhanced diagnostic clarity. Pathogen detection rates are presented in **Figure 4**.

### 3.5 Positivity with prior antibiotic exposure

**Figure 5:**
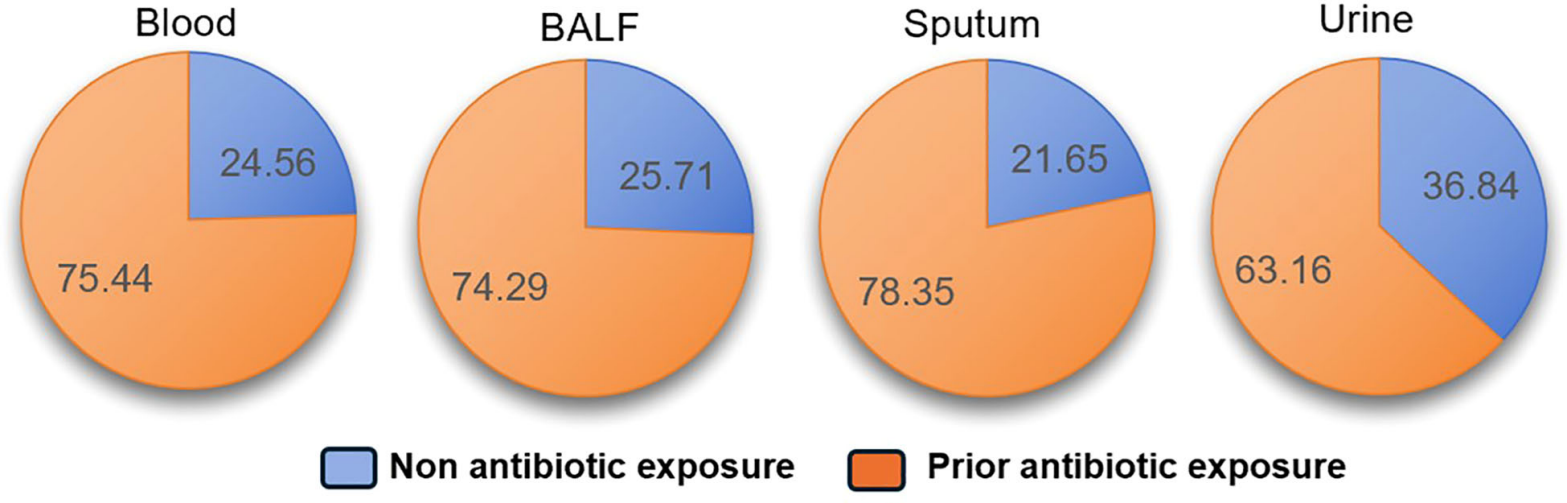
The proportion of prior antibiotic exposure among different sample types, expressed as percentages (%) for each group.

Among 243 kidney transplant recipients, 183 (75.31%) had received antimicrobial therapy prior to sample collection. The antibiotic exposure rate by sample type was highest in sputum (78.35%), followed by blood (75.44%), BALF (74.29%), and urine (63.16%) (**Figure 5**).

**Figure 6:**
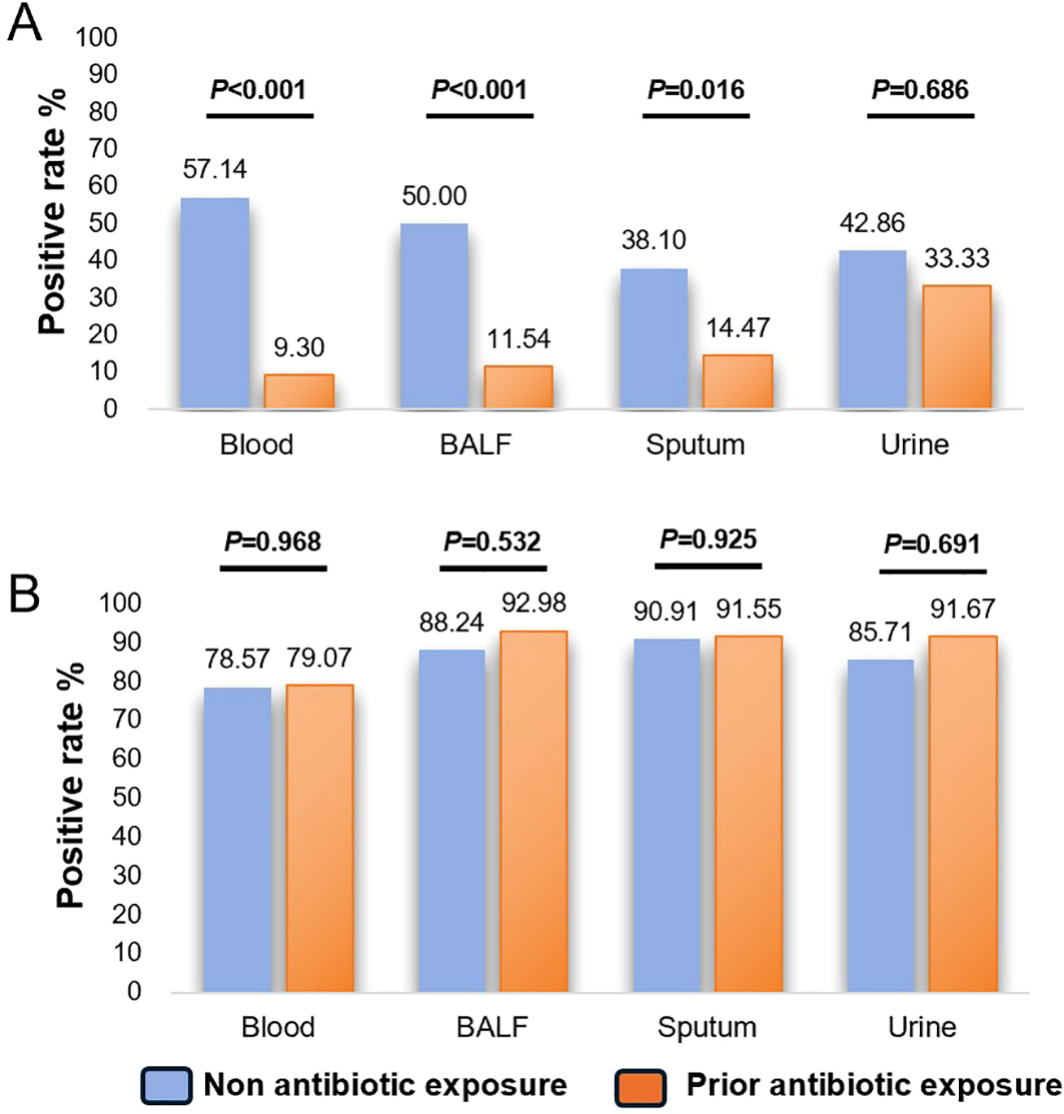
Positvity of prior antibiotic exposure. **(A)** Effect of Prior Antibiotic Exposure on the Positivity Rate of CMT for each sample. **(B)** Effect of Prior Antibiotic Exposure on the Positivity Rate of mNGS for each sample.

To evaluate the impact of prior antibiotic use, recipients were categorized into antibiotic pre-exposed group and non-exposed groups. mNGS demonstrated consistently high positivity regardless of prior antimicrobial exposure with no statistically significant difference between the two groups in blood (79.07% vs 78.57%, *P* = 0.968), BALF (92.98% vs 88.24%, *P* = 0.532), sputum (91.55%, vs 90.91%, *P* = 0.925, and urine (91.67% vs 85.71%, *P* = 0.691). Conversely, CMT performance was significantly compromised by antibiotic exposure, with detection rates markedly lower than in the non-exposed group via blood (9.3% vs 57.14%, *P* < 0.001), BALF (11.54% vs 50%, *P* < 0.001) and sputum (14.47% vs 38.10%, *P* = 0.016), but there was no significant difference was found in urine (33.33% vs 42.86%, *P* = 0.686) (**Figure 6**). These findings demonstrated that mNGS is less affected by prior antibiotic exposure, and may offer support its utility as a robust diagnostic tool for infection management in kidney transplant recipients.

### 3.6 Consistency and differences between mNGS and CMT detection results

**Figure 7:**
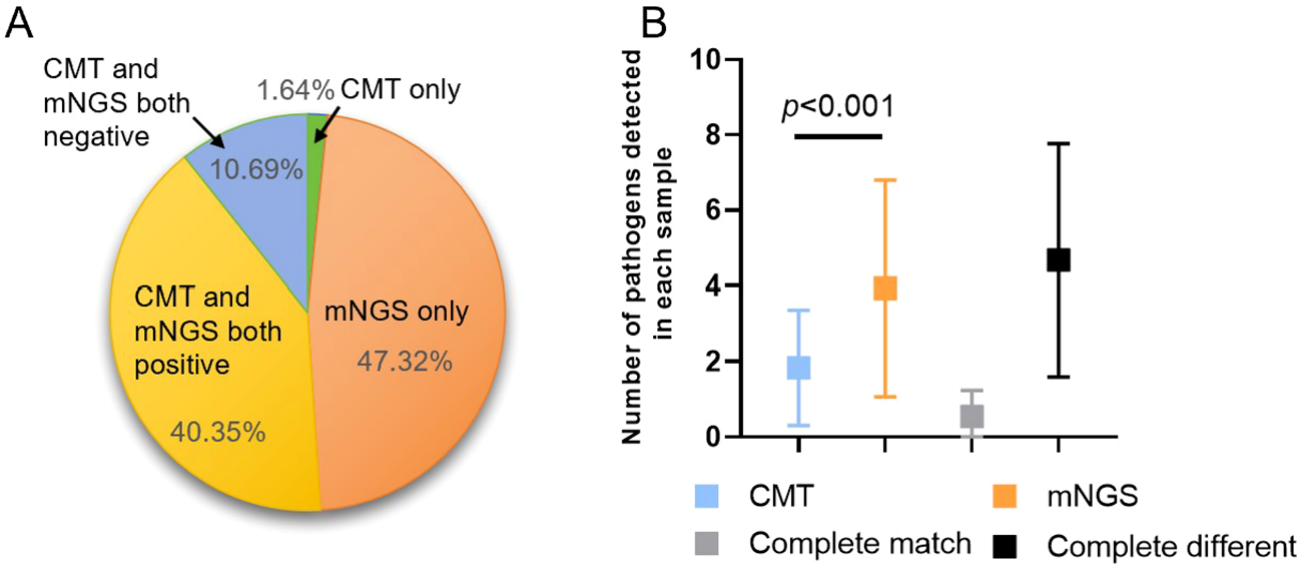
The consistency of CMT and mNGS. **(A)** The proportion of positive results in CMT and mNGS. **(B)** In double-positive results, the number of pathogens detected by CMT and mNGS.

In our study, 98 samples (40.35%) were positive by both mNGS and CMT. In these concordant samples, CMT identified a total of 178 pathogens, whereas mNGS identified 382 pathogens. The numbers of pathogens detected by mNGS and CMT were 3.93 ± 2.86 and 1.83 ± 1.53, with a significantly differences (*P* < 0.001) (**Figure 7**). Among these, CMT identified 56 samples of single pathogen infection, while mNGS identified 16 samples of single pathogen infection. In the 98 samples, only 3 samples showed complete concordance between the two methods, whereas the remaining 13 samples demonstrated completely discordant results. In each sample, the number of pathogens detected with complete concordance was 0.54 ± 0.69, whereas the number of completely discordant pathogens was 4.68 ± 3.08. A total of 43 samples showed that all pathogens detected by CMT were also identified by mNGS. The concordant pathogens included Stenotrophomonas maltophilia, Klebsiella pneumoniae, Pseudomonas aeruginosa, Acinetobacter baumannii, Escherichia coli, Haemophilus influenzae, Staphylococcus aureus, Candida krusei, Candida albicans, and Aspergillus fumigatus. In contrast, several pathogens, including TTV, CMV, SARS-CoV2, EBV, HSV-1, Enterococcus faecium, Pneumocystis jirovecii, Strongyloides stercoralis, Toxoplasma gondii were not detected by CMT.

Additionally, 146 samples (60.1%) were negative by CMT, whereas 211 samples (86.83%) were positive by mNGS. Interestingly, 4 samples (1.65%) were positive by CMT but negative by mNGS. A total of 26 samples (10.69%) yielded negative results by both methods. Based on mNGS results, 60 samples (24.69%) were classified as having single pathogen infections, while 151 samples (62.14%) were identified with polymicrobial infections. Among the 60 single pathogens identified by mNGS samples, 16 were also positive by CMT, including 3 samples with complete concordance and 13 samples with discordant results. Among the 151 mNGS identified polymicrobial infections, 77 samples were also positive by CMT. Of these, 40 samples showed that all pathogens identified by CMT were also detected by mNGS, whereas 37 samples demonstrated discordant pathogen profiles between the two methods. These findings indicate that pathogens detected by CMT were generally also captured by mNGS. In addition, mNGS is more effective in detecting viral infections as well as rare bacteria and parasites, which are difficult to identify by CMT. However, CMT was still able to culture pathogens that tested negative by mNGS, which may be attributed to the relatively small input volume (50 μL in each sample) used in mNGS, leading to potential false-negative results.

### 3.7 mNGS in clinical treatment decision

In this study, 183 of 243 recipients (75.31%) received anti-infective therapy prior to sample collection. Among them, 38 (21.1%) were treated with antibiotics alone, while the remainder received combination regimens involving antibacterial, antifungal, and/or antiviral agents. All recipients also underwent immunosuppressive adjustment, either through dose reduction or temporary discontinuation.

Based on the results of mNGS, targeted antimicrobial treatment was initiated in 60 recipients who had not received prior empirical therapy. Treatment selection was directly guided by pathogen identification. Among 183 recipients who had received prior antimicrobial exposure, 73 (39.89%) continued their existing anti-infective regimens without modification, whereas 110 (60.11%) required therapeutic adjustments, including escalation and de-escalation of antibiotic regimens or initiation of combination therapies. Specifically, 17 (9.3%) received modification of antibacterial therapy alone (including agent substitution or dose adjustment), 11 (6.01%) were switched to antiviral therapy alone, 7 (3.83%) received antifungal therapy alone, 32 (17.48%) received combined antibacterial and antifungal regimens, 7 (3.83%) received combined antibacterial and antiviral therapy, 36 (19.67%) were placed on triple therapy consisting of antibacterial, antifungal, and antiviral agents. mNGS results enabled de-escalation to narrower-spectrum antibiotics in 28 cases (15.23%) and reduced inappropriate antibiotic use in 33 cases (18.32%), particularly in patients with viral infections without bacterial or fungal co-infections. In addition, 11 cases (6.02%) of antibiotic-related adverse effects mainly include hematologic toxicity and gastrointestinal discomfort were improved following mNGS guided adjustment of antimicrobial therapy (**Table 2**).

**Table 2:** Adjustment of antibiotic treatment according to mNGS result.

Antibiotic regimen	Number of recipient n (%)
Antibacterial	17 (9.3%)
Antiviral	11 (6.01%)
Antifungal	7 (3.83%)
Antibacterial + Antifungal	32 (17.48%)
Antibacterial + Antiviral	7 (3.83%)
Antibacterial + Antifungal + Antiviral	36 (19.67%)
No change	73 (39.89%)
De-escalation to narrower-spectrum antibiotics	28 (15.23%)
Decrease antibiotic-related adverse effects	11 (6.02%)
Reduced inappropriate antibiotic use	33 (18.32%)

### 3.8 Drug resistance genes detected by mNGS

In our study, mNGS enabled the detection of antimicrobial resistance (AMR) genes in three clinical sample types: urine, sputum, and BALF. The majority of AMR positive samples were from sputum, followed by BALF and urine. Klebsiella pneumoniae was the most frequently identified resistance carrying pathogen and was detected across all three sample types. Escherichia coli and Acinetobacter baumannii were also common. Escherichia coli was identified in urine and sputum while Acinetobacter baumannii was detected in sputum and BALF. Among resistance genes, TEM was the most frequently identified (n=16), followed by CTX-M (n=7), SHV (n=7), OXA (n=6), and IMP (n=6) (**Table 3**). TEM, CTX-M, and SHV belong to the extended-spectrum β-lactamase (ESBL) family, which confers resistance to penicillins and third-generation cephalosporins in Gram-negative bacteria. OXA-type β-lactamases are commonly associated with carbapenem resistance in Acinetobacter baumannii and Pseudomonas aeruginosa whereas IMP is a Metallo-β-lactamase, mediates resistance to a broad range of β-lactam antibiotics, including carbapenems. The clinical relevance of identifying these genes lies in their capacity to guide precise antimicrobial selection and implement timely infection control interventions. By providing this genomic resistance profile in parallel with pathogen identification, mNGS offered significant diagnostic value beyond CMT and susceptibility testing, particularly in the setting of multidrug-resistant organisms.

**Table 3:** Antibiotic resistance gene.

Sample	mNGS result					
	Species	Drug class	Drug	DRG family	DRG	No
Urine	Klebsiella pneumoniae	Penicillins	Amoxicillin	SHV-type β-Lactamases	SHV_328	1
					SHV_1	1
		3rdGeneration Cephalosporins	Cefotaxime			
					SHV_2	1
	Escherichia coli	Penicillins	Amoxicillin	CTX-M-type ESBLs (Extended-Spectrum β-Lactamases)	CTX-M_69	1
					CTX-M_390	1
		3rdGeneration Cephalosporins	Cefotaxime			
					CTX-M_300	1
Sputum	Acinetobacter baumannii	Penicillins	Amoxicillin	OXA-type Carbapenemases (Oxacillinase)	OXA-23_114	1
					OXA-23_2	1
		Carbapenems	Imipenem		OXA-23_3	1
				TEM-type β-Lactamases	TEM_9	1
			Meropenem			
					TEM_151	1
		3rdGeneration Cephalosporins	Ceftazidime	IMP-type Metallo-β-Lactamases (MBLs)	IMP_6	1
					IMP_5	1
			Cefotaxime			
				VIM-type Metallo-β-Lactamases	VIM_15	1
	Pseudomonas aeruginosa	Carbapenems	Imipenem	TEM-type β-Lactamases	TEM_9	1
			Meropenem		TEM_151	1
		Penicillins	Amoxicillin	IMP-type Metallo-β-Lactamases (MBLs)	IMP_5	1
		3rdGeneration Cephalosporins	Cefotaxime	VIM-type Metallo-β-Lactamases	VIM_15	1
			Ceftazidime			
	Enterococcus faecium	Macrolides	Erythromycin	ErmB-type Ribosomal Methyltransferases	ErmB_233	1
		Lincosamides	Lincomycin		ErmB_8	1
	Staphylococcus aureus	Macrolides	Erythromycin	ErmB-type Ribosomal Methyltransferases	ErmB_485	1
					ErmB_268	1
		Lincosamides	Lincomycin			
				ErmC-type Ribosomal Methyltransferases	ErmC_8	1
	Streptococcus pneumoniae	Macrolides	Erythromycin	ErmB-type Ribosomal Methyltransferases	ErmB_5	1
		Lincosamides	Lincomycin		ErmB_56	1
	Klebsiella pneumoniae	Penicillins	Amoxicillin	CTX-M-type ESBLs (Extended-Spectrum β-Lactamases)	CTX-M_20	1
					CTX-M_713	1
					CTX-M_490	1
		3rdGeneration Cephalosporins	Cefotaxime	TEM-type β-Lactamases	TEM_187	1
					TEM_24	1
	Proteus mirabilis	Penicillins	Amoxicillin	SHV-type β-Lactamases	SHV_1	1
		3rdGeneration Cephalosporins	Cefotaxime	TEM-type β-Lactamases	TEM_24	1
			Ceftazidime			
	Escherichia coli	Penicillins	Amoxicillin	TEM-type β-Lactamases	TEM_35	1
					TEM_187	1
		3rdGeneration Cephalosporins	Cefotaxime		TEM_484	1
				CTX-M-type ESBLs (Extended-Spectrum β-Lactamases)	CTX-M_490	1
	Haemophilus influenzae	Penicillins	Amoxicillin	TEM-type β-Lactamases	TEM_410	1
		3rdGeneration Cephalosporins	Ceftazidime		TEM_110	1
			Cefotaxime			
BALF	Acinetobacter baumannii	Penicillins	Amoxicillin	OXA-type Carbapenemases (Oxacillinase)	OXA-23_5	1
		Carbapenems	Imipenem		OXA-23_29	1
			Meropenem		OXA-23_113	1
		3rdGeneration Cephalosporins	Cefotaxime	TEM-type β-Lactamases	TEM_243	1
			Ceftazidime			
	Enterobacter asburiae	Carbapenems	Imipenem	IMP-type Metallo-β-Lactamases (MBLs)	IMP_4	1
			Meropenem		IMP_6	1
		3rdGeneration Cephalosporins	Cefotaxime	KPC-type Serine Carbapenemases	KPC_187	1
			Ceftazidime		KPC_188	1
		Penicillins	Amoxicillin	SHV-type β-Lactamases	SHV_168	1
					SHV_79	1
				TEM-type β-Lactamases	TEM_289	1
					TEM_161	1
				VIM-type Metallo-β-Lactamases	VIM_30	1
					VIM_76	1
				NDM-type Metallo-β-Lactamases	NDM_366	1
	Klebsiella pneumoniae	Penicillins	Amoxicillin	IMP-type Metallo-β-Lactamases (MBLs)	IMP_4	1
		3rdGeneration Cephalosporins	Ceftazidime	KPC-type Serine Carbapenemases	KPC_187	1
			Cefotaxime	NDM-type Metallo-β-Lactamases	NDM_141	1
		Carbapenems	Imipenem	SHV-type β-Lactamases	SHV_168	1
			Meropenem	TEM-type β-Lactamases	TEM_289	1

## 4 Discussion

Kidney transplantation remains the preferred treatment for end-stage renal disease, with long term immunosuppressive therapy being essential to prevent allograft rejection. However, this immunosuppression markedly increases the risk of opportunistic infections, particularly pulmonary infections and urinary tract infections (UTIs), both of which are major contributors to allograft dysfunction, graft loss, and mortality in this population (Kinnunen et al., 2018). Although the gold standard for diagnosing these infections involves direct identification of the causative pathogens, clinical practice continues to rely heavily on CMT, such as culture and smear microscopy. These methods are limited by low sensitivity, prolonged turnaround times of up to 5 days or more longer, and susceptibility to interference from prior antimicrobial exposure often resulting in delayed or inaccurate diagnoses (Yang et al., 2024).

mNGS is an emerging technology that offers broad-spectrum, culture independent pathogen detection with a relatively rapid turnaround time, with results available within 24–48 hours. Unlike conventional methods, mNGS is less influenced by previous antimicrobial use and can simultaneously detect bacteria, viruses, fungi, parasites, and even antimicrobial resistance genes (Lv et al., 2024). These features make mNGS particularly valuable in immunocompromised populations, including kidney transplant recipients, where early and accurate pathogen identification is critical for guiding appropriate therapy and improving clinical outcomes (Zhao et al., 2024). However, the high cost of mNGS limits its application. In our center, the ratio of the cost of a single mNGS test to CMT is about 12: 1.

In our study, 54.73% of kidney transplant recipients experienced infections within the first three years posttransplant, with a disproportionately high infection rate (89.71%) observed among deceased donor recipients. These findings highlight the urgent need for enhanced diagnostic strategies in this high-risk group. Across the four sample types analyzed, Sputum samples yielded the highest mNGS positivity rate (92.71%), followed by BALF (90.14%), urine (89.47%), and blood (78.95%). The predominant detected pathogens included TTV, CMV, HSV-1, EBV, JCV, BKV, SARS-CoV-2, Pneumocystis jirovecii, Aspergillus fumigatus, Acinetobacter baumannii, Escherichia coli, Klebsiella pneumoniae, Pseudomonas aeruginosa and Enterococcus species which were found across multiple sample types. Among these, Escherichia coli and Klebsiella pneumoniae emerged as the most frequently isolated Gram-negative bacteria in infected patients. This finding is consistent with the study by Pinchera et al., which reported that the urinary tract is particularly susceptible to bacterial infection, with Gram-negative organisms accounting for more than 70%, and Escherichia coli identified as the most common isolate pathogen (Pinchera et al., 2024). Similarly, a study conducted in Nepal by Bikash Khatri et al. showed Escherichia coli again as the predominant causative pathogen (Khatri et al., 2022). Furthermore, other Gram-negative pathogens including Klebsiella pneumoniae, Pseudomonas aeruginosa and Enterococcus species are also recognized as important etiologic agents of UTIs in kidney transplant recipients, which was similar to the results of our study (McAteer and Tamma, 2024). In addition to bacterial pathogens, JCV was detected across three sample types urine (42.1%), blood (3.5%), and BALF (1.4%) whereas BKV was identified exclusively in urine samples, with a notably high detection rate of 73.68%. These viruses commonly establish latent infections in healthy adults (Keykhosravi et al., 2022; Sharma and Abdulkhalek, 2022), they may reactivate under immunosuppressive conditions and cause nephropathy in kidney transplant recipients. Differentiating between JCV and BKV remains challenging due to their similar cytopathic features, including identical viral inclusions and shared SV40 immunohistochemical staining positivity (Udomkarnjananun and Iampenkhae, 2023). Despite these challenges, both viruses were frequently detected in urine, A similar observation was seen in previous study which indicating that JCV and BKV appear earlier in urine than in blood (Demey et al., 2024; Tomb et al., 2025),highlighting its diagnostic value in suspected UTIs. Surprisingly, the impact of prior antibiotic exposure on pathogen detection was assessed for both CMT and mNGS. mNGS was less affected by prior antibiotic use compared with CMT. However, no significant difference was observed for urine based CMT. When considering factors such as prevalent pathogens and cost-effectiveness, CMT may still offer practical advantages, particularly when urine samples are used for the diagnosis of UTIs in kidney transplant recipients. Moreover, TTV is increasingly recognized as a surrogate marker for immune status. Elevated TTV loads may reflect heightened susceptibility to opportunistic infections and increased risk of allograft rejection (Querido et al., 2023; Cabezas et al., 2024; Reyes et al., 2024), While CMV continues to be a major infectious complication posttransplant and is routinely included in prophylactic protocols (Limaye et al., 2023; Grossi and Peghin, 2024; Stewart and Kotton, 2024). In our study, TTV and CMV were detected at high rates by mNGS but not identified by CMT. Given the inherent challenges in viral detection using routine culture-based methods. Additionally, among fungal pathogens, Pneumocystis jirovecii and Aspergillus fumigatus were the most commonly identified species, this findings agree with other reports, which similarly identified them as the main cause of fungal pulmonary infection in kidney transplant recipients (Wilmes et al., 2021). Pneumocystis jirovecii is a common opportunistic fungal pathogen that poses a significant risk in this population, and prophylaxis with trimethoprim-sulfamethoxazole (TMP-SMX) within the first 6 months after transplantation is recommended to reduced its incidence (Eberl et al., 2024; Zhou et al., 2024).In this present study, mNGS detected Pneumocystis jirovecii in 7.02% of blood samples and 9.38% of sputum samples emphasizing the diagnostic value of both respiratory and blood samples for its detection in kidney transplant recipients. Similarly, Aspergillus fumigatus was identified across multiple sample types including blood, sputum, and BALF samples, demonstrating its prevalence as an opportunistic fungal pathogen. Unlike CMT, which often fails to detect fungal organisms unless invasive procedures are performed (Wilmes et al., 2021). mNGS demonstrated the superior ability to detect pathogens even in recipient who had received prior antibiotic therapy, as evidenced by our study, in which the overall positivity rate of mNGS was significantly higher than that of CMT. Among 243 samples, only 98 samples were positive by both mNGS and CMT, whereas 146 samples (60.1%, 146/243) were negative by CMT and 211 samples (86.8%, 211/243) were positive by mNGS. The most common pathogens detected by CMT also identified by mNGS included Stenotrophomonas maltophilia, Klebsiella pneumoniae, Pseudomonas aeruginosa, Acinetobacter baumannii, Escherichia coli, Haemophilus influenzae, Staphylococcus aureus, Candida krusei, Candida albicans, and Aspergillus fumigatus. Notably, no viral or parasitic pathogens were detected by CMT. Collectively, these findings highlight the pivotal role of mNGS in pathogen detection for identifying clinically relevant viral and fungal infections and facilitating timely assessment of immune status in kidney transplant recipients.

In addition to its broad-spectrum pathogen detection capabilities, mNGS can also identify AMR genes. In our study, a total of 58 resistance genes across 10 AMR gene families were detected across urine, sputum, and BALF samples. The most prevalent detected antimicrobial resistance genes were from the ESBL families and carbapenemase families including TEM, SHV, CTX-M and OXA that confer resistance to penicillins, third-generation cephalosporins, and carbapenems, thereby complicating empirical treatment decisions (Lepe and Martínez-Martínez, 2022). These genes are commonly associated with Gram-negative organisms such as Escherichia coli and Klebsiella pneumoniae (TEM, CTX-M, SHV) (Tamma et al., 2021), as well as Acinetobacter baumannii or Pseudomonas aeruginosa (OXA) (Boyd et al., 2022)which were also among the most common pathogens in our study. Acinetobacter baumannii is a well- recognized nosocomial pathogen capable of causing severe infections including pneumonia, bloodstream infections, and urinary tract infections. It presents a significant global health threat due to its high rates of multidrug resistance (MDR), which making treatment increasingly challenging. This organism often exhibits resistance to multiple antibiotic classes, including β-lactams such as carbapenems, penicillins, and third-generation cephalosporins, fluoroquinolones, aminoglycosides, and even colistin. Carbapenem-resistant Acinetobacter baumannii (CRAB) is designated as a critical priority pathogen by the World Health Organization (Muller et al., 2023; Shadan et al., 2023), underscoring the urgent need for enhanced diagnostics, surveillance, and treatment strategies.

Benefiting from its short turnaround time, broad spectrum pathogen detection, and the ability to identify antimicrobial resistance genes, mNGS provided complementary guidance for clinical decision making. In the present study, the mean length of hospital stay for all recipients was 26.48 ± 22.72 days. A total of 40 recipients (16.46%) required ICU admission, with a mean ICU stay of 37.30 ± 32.42 days. In most cases, mNGS was performed after recipients failed to achieve clinical improvement following anti-infective treatment based on CMT findings. At the time of admission, 230 recipients exhibited abnormal chest CT findings, including 223 cases with bilateral lesions and 7 cases with unilateral lesions. The predominant imaging features consisted of scattered patchy opacities and ground glass opacities. kidney function impairment was observed in all recipients, as reflected by elevated serum creatinine levels, particularly among those requiring ICU care, in whom the mean creatinine level was 108.39 ± 225.22 μmol/L. Notably, 60.11% of recipients had their treatment regimens adjusted based on mNGS results, thereby reducing unnecessary antibiotic exposure and minimizing antibiotic-associated adverse effects. Specifically, regimen modifications included antibacterial therapy in 17 cases (9.3%, 17/183), antiviral therapy in 11 cases (6.01%, 11/183), and antifungal therapy in 7 cases (3.83%, 7/183). In addition, combination therapy was implemented in 32 cases (17.48%, 32/183) with antibacterial plus antifungal agents, in 7 cases (3.83%, 7/183) with antibacterial plus antiviral agents, and in 36 cases (19.67%, 36/183) with triple therapy comprising antibacterial, antifungal, and antiviral agents. The majority of these recipients achieved positive clinical outcomes, as evidenced by resolution of bilateral patchy pulmonary opacities compared with admission imaging and a decline in serum creatinine levels toward baseline values. Only one recipient died from infection, in this case, an approximately three-week delay occurred between hospital admission and the initial mNGS testing. During this interval, empirical broad spectrum anti-infective therapy, including antiviral, antibacterial, and antifungal agents, was initiated based on inflammatory markers and conventional culture results. However, despite multiple escalations of antibacterial therapy, the recipient’s clinical condition failed to improve. This limited response may be attributed to a restricted capacity to detect polymicrobial infections. In contrast, mNGS provides a broad spectrum of pathogens including bacteria and viruses in a single assay, while also offering information on relative pathogen sequence read abundance. Following optimization of the anti-infective regimen based on mNGS results, a short-term clinical response was observed, characterized by a gradual increase in the oxygenation index and progressive reductions in inflammatory markers and body temperature, indicating an initial therapeutic response. The temporary clinical improvement following mNGS guided antimicrobial adjustment supports the clinical utility of mNGS in the etiological diagnosis and management of complex infections in kidney transplant recipients. Nevertheless, despite pathogen identification and targeted antimicrobial adjustments, definitive treatment success was not achieved, largely attributable to the recipient’s profound underlying immunosuppression. The severely impaired host immune response likely limited the effectiveness of antimicrobial therapy, while the emergence of additional pathogens during the later course of disease further complicated clinical management. Continuous polymicrobial invasion, superimposed on marked immune dysfunction, ultimately led to irreversible clinical deterioration. Notably, the carbapenemase resistance gene IMP-5 and several multidrug-resistant organisms including Klebsiella pneumoniae, Pseudomonas aeruginosa and Acinetobacter baumannii, as well as TTV and human herpesvirus type 5 (HHV-5) which are not detectable by CMT were detected in this case. The presence of these resistance determinants and viral co-infections may have contributed to the poor therapeutic response, further underscoring the complexity, rapid progression, and high mortality risk associated with severe infections in kidney transplant recipients. Although most recipients achieved clinical improvement, with only one death reported, it should be noted that the study population included recipients who had failed CMT guided treatment or experienced clinical deterioration. Additionally, the mean interval of 3.22 ± 2.81days between CMT and mNGS testing complicates the attribution of clinical outcomes to mNGS. Most recipients had already received empirical therapy based on CMT results prior to mNGS testing, and thus clinical outcomes may have been influenced by the delayed response to initial CMT guided treatment combined with mNGS guided antimicrobial adjustments. Consequently, it is difficult to determine the extent to which observed outcomes can be directly attributed to mNGS.

Despite the demonstrated diagnostic potential of mNGS in infectious diseases, its widespread clinical adoption remains constrained by challenges related to result interpretation and ethical considerations. Kidney transplant recipients frequently exhibit latent viral reactivation (Cherneha et al., 2021; Asif et al., 2024) or low-level microbial DNA that may not represent true invasive disease (Chen et al., 2024). In this context, mNGS cannot always distinguish whether detected sequences originate from colonizing organisms or true causative pathogens (Yi et al., 2024; Zhao et al., 2024).In the absence of a standardized interpretative framework, the detection of multiple potential pathogens further complicates causal attribution and may increase the risk of over-treatment (Elbehiry and Abalkhail, 2025). Additional concerns arise when mNGS results are discordant with those of CMT. These conflicting results may generate diagnostic uncertainty, potentially increasing anxiety among both patients and clinicians. This uncertainty can complicate clinical decision-making and underscores the importance of interpreting mNGS findings within the broader clinical, microbiological, and epidemiological context (Marra et al., 2024). From an ethical and health economic perspective, the substantially higher cost of mNGS raises important questions regarding proportionality and equitable resource allocation, particularly in the setting of finite healthcare resources (Ibrahim et al., 2026). In resource limited environments, allocation of funds toward universal high-cost diagnostics may result in opportunity costs, potentially diverting resources from preventive strategies, antimicrobial stewardship programs, or long-term transplant care. Although several studies have reported the diagnostic yield of mNGS in kidney transplant populations, the absence of formal cost-effectiveness, cost-utility, budget impact, and quality-adjusted life-year analyses limit the ability to guide rational policy decisions (Tian et al., 2022; Huang et al., 2024; Ye et al., 2024). As noted in our study, the cost ratio between mNGS and CMT was approximately 12:1. However, this figure alone does not adequately reflect the true economic value of mNGS in clinical practice. Without comprehensive economic evaluation, it is difficult to ensure that technological advancement aligns with both demonstrable clinical benefit and ethical resource allocation. Given these barriers, despite its advantages as a rapid and broad-spectrum pathogen detection tool, the routine use of mNGS as part of universal dual-testing strategies remains difficult to justify.

Lastly, Due to the immunosuppressed status of kidney transplant recipients, there is an urgent need for rapid and comprehensive diagnostic approaches to guide precise clinical management. In this study mNGS achieved significantly higher pathogen detection rates across all sample types and enabled the identification of rare, fastidious, and difficult to culture organisms that are frequently missed by CMT. In addition, mNGS provided important information on AMR genes, thereby facilitating more timely and targeted antimicrobial therapy. These findings suggest that mNGS may serve as a valuable adjunct to CMT in kidney transplant recipients with suspected pulmonary or urinary tract infections Our findings further support the complementary role of sputum and BALF mNGS alongside CMT in recipients with pneumonia, particularly in cases with negative conventional culture results or in recipient who experience clinical deterioration despite empiric treatment. In contrast, for urinary tract infections, urine CMT may remain the preferred initial diagnostic approach, considering pathogen profiles, turnaround time, and cost-effectiveness. Therefore, mNGS should be used selectively according to clinical symptoms and manifestations.

## 5 Limitations

While mNGS offers considerable benefits in the detection of infectious pathogens, there were several limitations in our study, First, the retrospective design and absence of a control group limit causal inference. All included recipients underwent both CMT and mNGS testing, which precludes differentiation of the independent clinical impact attributable to each modality. As a result, the relative contribution of CMT and mNGS to diagnostic performance, treatment modification, and clinical outcomes cannot be determined. Second, the relatively small sample size may limit the strength of statistical inference and generalizability of the findings. Larger, multicenter studies are therefore warranted to validate these results and to assess long-term clinical outcomes in diverse transplant populations. Third, the lack of a comprehensive cost analysis prevents assessment of the economic implications of mNGS implementation. Without formal cost-effectiveness evaluation, it is not possible to establish the superiority or economic justification of any specific diagnostic algorithm. Implementation in routine clinical practice should be considered only after robust evidence establishes clear patient-centered benefit and support responsible resource allocation. Therefore, prospective comparative studies that include distinct patient cohorts tested exclusively with CMT or exclusively with mNGS, randomized controlled trials or step-wedge implementation designs are needed to determine whether selective deployment of mNGS improves clinically meaningful outcomes while ensuring responsible resource utilization. Importantly, these prospective designs should incorporate standardized clinical endpoints (e.g., mortality, length of hospital stay, infection-related complications, readmission rates) alongside detailed analyses of antimicrobial stewardship (e.g., antibiotic consumption, de-escalation rates, adverse effects). From a health economics perspective, integrating formal cost-effectiveness analyses into these studies will be essential to determine whether selective deployment of mNGS provides measurable patient-centered benefits while ensuring responsible resource allocation. Given these limitations, recommending mNGS as a routine first-line diagnostic test for infectious diseases in kidney transplant recipients remains challenging, instead, it may be better positioned as a supplementary tool alongside conventional microbiological testing.

## 6 Conclusion

In summary, mNGS offers significant advantages by providing an efficient, rapid, and relatively comprehensive diagnostic approach for infections in kidney transplant recipients. In complex, severe, or refractory infections especially in such scenarios where CMT unable to definitively exclude infection or yields negative results or when the patient’s condition deteriorates despite ongoing treatment, mNGS may serve as a valuable adjunctive tool for pathogen detection and assist in guiding the selection of appropriate antimicrobial therapy. However, given the relatively high cost of mNGS, its use should be carefully individualized according to specific clinical contexts to ensure diagnostic accuracy.

## Data Availability

The data presented in the study are deposited in the NCBI BioProject repository, accession number PRJNA1494152.
